# Simultaneous 11C-Methionine Positron Emission Tomography/Magnetic Resonance Imaging of Suspected Primary Brain Tumors

**DOI:** 10.1371/journal.pone.0167596

**Published:** 2016-12-01

**Authors:** Cornelius Deuschl, Sophia Goericke, Johannes Grueneisen, Lino Morris Sawicki, Juliane Goebel, Nicolai El Hindy, Karsten Wrede, Ina Binse, Thorsten Poeppel, Harald Quick, Michael Forsting, Joerg Hense, Lale Umutlu, Marc Schlamann

**Affiliations:** 1 Institute of Diagnostic and Interventional Radiology and Neuroradiology, University Hospital Essen, Essen, Germany; 2 Erwin L. Hahn Institute for Magnetic Resonance Imaging, University of Duisburg-Essen, Essen, Germany; 3 Institute of Diagnostic and Interventional Radiology, University Hospital Duesseldorf, Duesseldorf, Germany; 4 Department of Neurosurgery, University Hospital Essen, Essen, Germany; 5 Department of Nuclear Medicine, University Hospital Essen, Essen, Germany; 6 High Field and Hybrid MR Imaging, University Hospital Essen, Essen, Germany; 7 Department of Medical Oncology, West German Cancer Center, University Hospital Essen, Essen, Germany; 8 Department of Neuroradiology, University Hospital Giessen, Gießen, Germany; Wayne State University, UNITED STATES

## Abstract

**Introduction:**

The objective of this study was to assess the diagnostic value of integrated 11C- methionine PET/MRI for suspected primary brain tumors, in comparison to MRI alone.

**Material and Methods:**

Forty-eight consecutive patients with suspected primary brain tumor were prospectively enrolled for an integrated 11C-methionine PET/MRI. Two neuro-radiologists separately evaluated the MRI alone and the integrated PET/MRI data sets regarding most likely diagnosis and diagnostic confidence on a 5-point scale. Reference standard was histopathology or follow-up imaging.

**Results:**

Fifty-one suspicious lesions were detected: 16 high-grade glioma and 25 low-grade glioma. Ten non-malignant cerebral lesions were described by the reference standard. MRI alone and integrated PET/MRI each correctly classified 42 of the 51 lesions (82.4%) as neoplastic lesions (WHO grade II, III and IV) or non-malignant lesions (infectious and neoplastic lesions). Diagnostic confidence for all lesions, low-grade astrocytoma and high-grade astrocytoma (3.7 vs. 4.2, 3,1 vs. 3.8, 4.0 vs. 4,7) were significantly (p < 0.05) better with integrated PET/MRI than in MRI alone.

**Conclusions:**

The present study demonstrates the high potential of integrated 11C-methionine-PET/MRI for the assessment of suspected primary brain tumors. Although integrated methionine PET/MRI does not lead to an improvement of correct diagnoses, diagnostic confidence is significantly improved.

## Introduction

Diagnosis of primary brain tumors and other focal intracranial mass lesions based on magnetic resonance imaging (MRI) imaging is still challenging. MRI, the gold standard for oncologic imaging of the brain, gives detailed morphologic imaging aspects and is indispensable for diagnosing suspected primary brain tumors. Structural MRI sequences including susceptibility weighted imaging, pre-/ and post gadolinium -T1 and T2 fluid-attenuated inversion recovery (FLAIR), preferentially as high-resolution 3D-sequences, are essential for diagnosis, preoperatively planning and intraoperative navigation [1,2]. In contrast, positron emission tomography (PET) offers insight into brain tumor pathophysiology and metabolic processes depending on the chosen tracer. 11C-methionine is associated with protein synthesis; it illustrates the transmembrane transport by the sodium independent amino acid L-transporter into cells [3]. 11C-methionine PET has been shown to have high sensitivity and high specificity for diagnosing primary brain tumor [4,5]. Methionine PET was proven helpful for detecting glial tumors by showing elevated methionine uptake, although not all glial tumors show uptake [6,7,8]. Recent publication showed that methionine-PET provides information on patient prognosis [9,10]. Nevertheless treatment decision especially in non-enhancing lesions mimicking low-grade glioma is challenging and not yet solved. Up to now many studies have been conducted in order to describe either metabolic pattern of focal brain lesions by methionine-PET or pure morphologic information by MRI, lacking a dedicated study on the diagnostic value of integrated methionine-PET/MRI. Integrated PET/MRI scanners provide simultaneous morphologic and metabolic information with an excellent co-registration in a single hybrid examination, rendering this hybrid imaging modality available for an easy and broad clinical application.

Our purpose was to evaluate the diagnostic value of integrated methionine-PET/MRI in the diagnostic work-up of suspected primary brain tumors.

## Material and Methods

### Patients and study inclusion criteria

The study was conducted in conformance with the Declaration of Helsinki and approved by the Ethics Commission of the Medical Faculty of the University Duisburg-Essen (study number 11–4822-BO). All patients gave written informed consent before undergoing 11C-methionine PET/MRI. Patients were assigned to PET/MRI when suspected primary brain tumor in MRI scan. Overall 48 patients (24 women, 24 men, mean age 35.1 years, range 19.8–70.5 years) with suspected primary brain tumor were assigned to integrated 11C-methionine PET/MRI.

#### Reference standard

Histopathological confirmation was available in 28 patients. In the remaining 20 patients the suspected pathology was not confirmed histopathologically, because lesion location was either in an eloquent region, patient denied biopsy/surgery or the lesion was diagnosed as non malignant. Therefore, when histopathologic confirmation was not available, only patients with MRI imaging-follow-up of at least one year were included in the study. MRI was used as the reference standard in 20 Patients (12 patients with LGG and 8 patients with non malignant lesions). In patients without histopathological confirmation the final score for each lesion was assessed in a consensus reading by two experienced neuroradiologists (12 and 14 years of experience) for the determination of the reference standard. Imaging-based differentiation of a lesion is less solid than histopathological diagnosis. Therefore remains a residual uncertainty in these lesions without histopathological confirmation.

### PET/MR Imaging

All PET/MR imaging examinations were performed on a 3-Tesla Biograph mMR (Siemens Healthcare, Erlangen, Germany), whole-body hybrid imaging system. The fully integrated PET detector in the iso-center of the MR system consists of 8 detector rings with each 56 lutetium oxyorthosilicate scintillator crystal blocks, read out by MR-compatible avalanche photodiodes, and provides a PET imaging FOV of 25.8 cm in axial direction [11], [12].

Patients with suspected primary brain tumors underwent PET/MR imaging after injection of 11C-methionine (mean ± SD, 981 MBq; ± 210 MBq; range, 300–1200 MBq). Before tracer administration, a fasting period of at least 4 hours was assured. PET/MR imaging scans started at an average delay of 22.2 ± 4.7 min, range 10–37 min after intravenous methionine administration.

A dedicated 16-channel head and neck radiofrequency coil was used for MR imaging. MR imaging was performed simultaneously to PET data acquisition.

The MRI protocol included an transversal weighted T1-weighted-TIRM-Dark-Fluid-sequence (repetition time [TR], 2000 ms; echo time [TE], 13 ms; slice thickness (ST), 5 mm), 3D-FLAIR-sequence (TR 5000 ms, TE 395 ms, ST 1 mm) with 3D-reconstructions, a transversal diffusion-weighted-imaging-sequence (DWI) (TR 7900 ms; TE 101 ms; diffusion weighting (b-values), 0, and 1,000 s/mm2; ST 5 mm), a transversal susceptibility-weighted imaging-sequence (TR 26 ms, TE 20 ms, ST 2 mm), contrast enhanced 3D-MPRAGE in sagittal orientation (TR 1790 ms, TE 2.67 ms, ST 1 mm, 1 mmol/10 kg body weight of contrast agent (Dotarem®, Guerbet, Sulzbach/Taunus, Germany) with 3D-reconstructions, contrast enhanced T1 in transversal orientation (TR 162 ms, TE 5.05 ms, ST 5 mm).

PET of the head was performed in one bed position (axial field of view 25.8 cm) with an acquisition time of 20 min while simultaneously acquiring the MRI sequences. The PET data were reconstructed in 3D mode using ordinary Poisson ordered subsets expectation maximization with 3 iterations and 21 subsets and a Gaussian filter with 4 mm FWHM and 344×344 voxels.

### Image analysis

Two neuro/radiologists with 12 and 4 years of experience in interpreting MR imaging and with 4 and 2 years of experience in interpreting hybrid imaging, respectively, rated the images separately in random order utilizing dedicated viewing software for integrated imaging (OsiriX, Pixmeo SARL, Bernex, Switzerland). Patient- and lesion-based image analysis was performed in two sessions separated by a minimum of 4 weeks to avoid recognition bias. The first session comprised interpretation of the MR imaging datasets alone. In the second session integrated PET/MRI datasets were assessed. Both readers were blinded to patient identification data and diagnosis and were asked to identify all suspected primary brain tumors and give the most likely diagnosis neoplastic lesions (astrocytoma WHO grade II, III and GBM, Oligodendroglioma) or non-malignant lesions (infectious, vascular, degenerative and unclear lesions). Suspected gliomas were also rated as low-grade (WHO° I and II) or high-grade glioma (WHO°III and IV). Furthermore diagnostic confidence for the given diagnosis on a five-point Likert scale (1—not at all confident, 2—not very confident, 3—neutral, 4—confident, 5—very confident) was assessed. In case of different scores between the raters, they came to a consensus after re-reviewing the images together.

In PET malignancy was considered if focal methionine uptake was visible. A lesion was assessed by standardized uptake value of the tumor showing the maximum uptake (SUVmax). For differentiation between tumor and non neoplastic lesions mean T/N 1,5 was used [13] and for differentiation between grades II and III SUVmax 2,36 was taken [14].

### Statistical Analysis

For statistical analysis SPSS version 21 (IBM) was used. Data are presented as mean ± standard deviation [15]. Descriptive analysis was used to evaluate the resulting scores. The Wilcoxon signed-rank test was used to indicate potential significant differences between MRI and integrated methionine PET/MRI datasets. Mann-Whitney-U-Test was used for group comparison to show potential significant differences. P values of less than 0.05 were considered statistically significant.

## Results

### Patient based analysis

Methionine-PET/MR imaging examinations were successfully completed for all 48 patients (100%).

In 16 patients high-grade glioma were found (3 glioblastoma multiforme (GBM), 8 astrocytoma WHO grade III, 2 oligodendroglioma WHO grade III, 3 oligoastrocytoma WHO grade III). All high-grade glioma were confirmed histopathologically.

Low-grade gliomas were detected in 24 patients (20 astrocytoma WHO grade II, 1 oligodendroglioma WHO grade II, 1 oligoastrocytoma WHO grade II and 2 ganglioglioma WHO grade I), whereas 12 gliomas were confirmed by histopathology and 12 gliomas by follow-up imaging. In one patient, two lesions were detected, what explains the divergent number of low-grade astrocytomas in the patient- and lesion-based analysis.

In eight patients suspicious lesions were assessed as non-malignant, of which five were rated as inflammatory disease and three as vascular disease. In two patients two lesions were detected, what explains the divergent number of vascular lesions in the patient- and lesion-based analysis. Reference standard for these lesions was follow-up imaging for at least one year.

### Lesion based analysis

**Fig 1:**
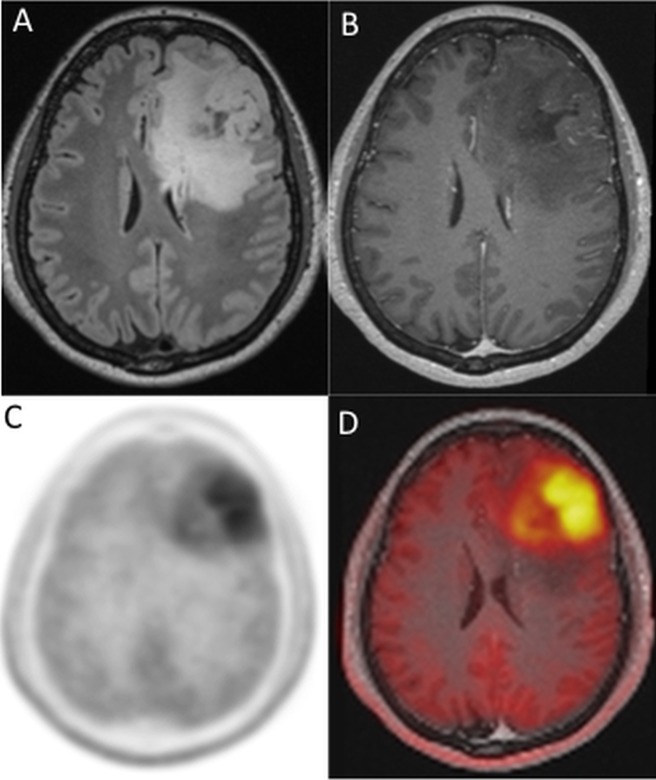
Astrocytoma WHO grade III. 20-year old woman with left frontal tumor with FLAIR- hyperintensity (A) and minimal contrast enhancement (B); leading to a diagnosis of an astrocytoma WHO grade III by MRI alone. In methionine-PET an intensive tracer more-uptake (T/N ratio 4.9) of this lesion is seen (C, D fused), making diagnosis of an astrocytoma WHO grade III with an oligodendroglial component more likely.

**Fig 2:**
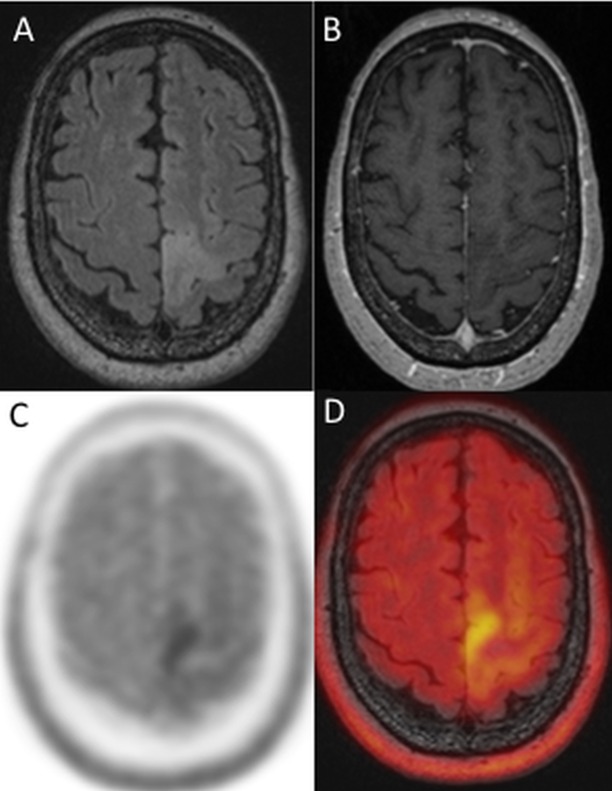
Astrocytoma WHO grade II. 71-year old female patient with left sided pre-/central FLAIR—hyperintensity (A) without contrast enhancement (B). Integrated methionine PET showed a focal pathologic tracer uptake (SUVmax 2.5 T/N, ratio 2.27) of this lesion (C, D (fusion of FLAIR and PET)). Diagnosis was an astrocytoma WHO grade II.

In all 48 patients a total of 51 suspicious lesions were seen. In MRI alone as well as in integrated methionine PET/MRI 42 of the 51 lesions (82.4%) were correctly described according to the reference standard (Table 1). Among those 51 lesions 41 were gliomas, of which 25 were low-grade and 16 high-grade gliomas (Figs 1 and 2). Methionine PET/MRI- and MRI-datasets alone did not vary in differentiating low-grade from high-grade glioma, both modalities correctly described 36 of 41 gliomas (87.8%). Methionine PET/MRI- and MRI-datasets alone did also not discern in differentiating non-malignant lesions from glioma.

**Table 1:** The data display diagnosis based on the reference standard for all lesions and for all high/low grade astrocytoma with structural MRI alone and with integrated 11C-methionine PET/MRI.

Procedure	Correct diagnosis all lesions	Incorrect diagnosis all lesions	Correct diagnosis: low-grade astrocytoma vs. high-grade astrocytoma	Incorrect diagnosis: low-grade astrocytoma vs. high-grade astrocytoma
	Number (%)	Number (%)	Number (%)	Number (%)
**MRI**	42/51 (82.4%)	9/51 (17.6%)	36/41 (87.8%)	5/41 (12.2%)
**PET/MRI**	42/51 (82.4%)	9/51 (17.6%)	36/41 (87.8%)	5/41 (12.2%)

**Fig 3:**
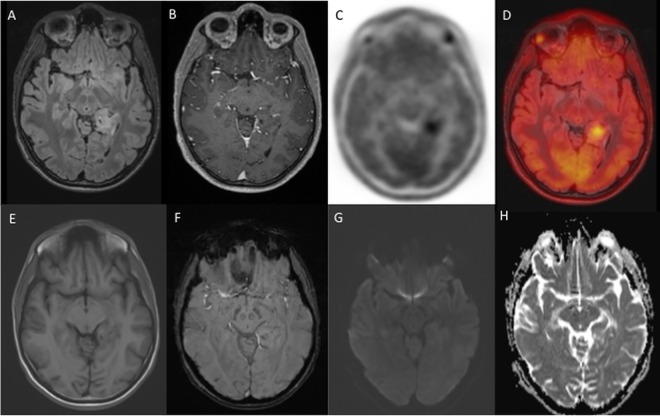
Astrocytoma grade II. 21-year old male patient with a left sided temporomesial, blurry demarked FLAIR-hyperintens lesion (A), without contrast-enhancement (B), but with focal methionine uptake (SUVmax 1.5, T/N ratio 1.9) (C, D (PET-fusion image with FLAIR-images)). No correlate of the lesion was found in native T1w (E), SWI (F), DWI-b1000 (G) and ADC-images (H). 10 months after initial methionine PET/MRI a progress from a formerly astrocytoma °2 to a high-grade glioma was suspected and operation finally revealed a histopathologically confirmed GBM.

One lesion was identified by MRI alone as an astrocytoma WHO grade III, whereas integrated methionine PET/MRI made an astrocytoma WHO grade III with an oligodendroglial component more likely due to the intense tracer uptake [13,16]; histopathological diagnosis confirmed an anaplastic oligodendroglioma WHO grade III (Fig 1). In another patient one lesion of the left mesial temporal lobe was detected in FLAIR-imaging, without contrast-enhancement, diffusion restriction or susceptibility artefacts in MRI alone (Fig 3), making the diagnosis of an astrocytoma WHO grade II. Integrated methionine-PET data showed an intensive tracer uptake of this lesion (SUVmax 1.5, T/N ratio 1.9) (Fig 3). Based on previous publications this astrocytoma has a likely poor prognosis due to the intensive traceruptake [9,10]. This patient did not want to go for surgery immediately. In external follow-up imaging 10 months later MRI showed a new contrast-enhancement, central necrosis and hemorrhage, indicative for a malignant transformation, and the final histopathological diagnosis was GBM.

Furthermore, 10 non-oncologic lesions were identified in 8 patients, of which 6 lesions were correctly determined on MRI alone and methionine PET/MRI (Table 2). These non-oncologic lesions were infectious (acute disseminated encephalomyelitis (ADEM), limbic encephalitis), vascular (cortical vein thrombosis, cavernoma), degenerative (Wallerian degeneration) and unclear (4 lesions most likely infectious, most likely ischemic).

**Table 2:** The data display diagnosis based on the reference standard for vascular, autoimmune and other lesions with structural MRI alone and with integrated 11C-methionine PET/MRI.

Procedure	Correct diagnosis for vascular, autoimmun or other lesions
	Number (%)
**MRI**	6/10 (60%)
**PET/MRI**	6/10 (60%)

Additional methionine PET data did not change clinical management in any patient.

#### Methionine PET

**Fig 4:**
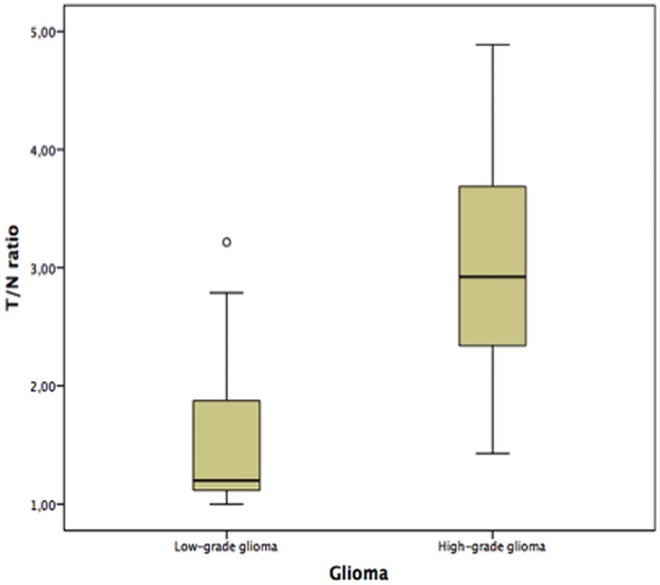
Boxplot of low-grade and high-grade glioma. Boxplot of the SUVmax T/N ratios of low-grade and high-grade glioma. Despite the means differed significantly both entities showed a broad overlap.

Methionine-PET showed a pathologic T/N ratio (T/N ratio >1.5) in 8/25 lesions (Fig 2) of low-grade glioma and 14/16 lesions of high-grade glioma. SUVmax T/N ratios of low-grade and high-grade glioma were 1.5 ± 0.61 and 2.9 ± 1.01, respectively. The difference was statistically significant (p < 0.001), but an obvious overlap between low-grade (n = 25) and high-grade glioma (n = 16) in Box-plot was prominent (Fig 4).

#### Diagnostic confidence

Diagnostic confidence for classification of all lesions reached significantly higher scores for methionine PET/MRI than for MRI alone (p < 0.05) (Table 3).

**Table 3:** Resulting scores for diagnostic confidence of all lesions.

Modality	MRI alone	Methionine PET/MRI
Mean ± SD	3.7 ± 1.04	4.2 ± 1.04
Median	4 (range 1–5)	5 (range 1–5)

Diagnostic confidence for diagnosis of all low-grade astrocytoma as well as for all high-grade astrocytoma between methionine PET/MRI and MRI alone were significantly higher with methionine PET/MRI (p < 0.05) (Tables 4 and 5).

**Table 4:** Resulting scores for diagnostic confidence of low-grade astrocytoma.

Modality	MRI alone	Methionine PET/MRI
Mean ± SD	3.1 ± 0.98	3.8 ± 1.24
Median	3 (range 1–4)	4 (range 1–5)

**Table 5:** Resulting scores for diagnostic confidence of high-grade astrocytoma.

Modality	MRI alone	Methionine PET/MRI
Mean ± SD	4.0 ± 0.75	4.7 ± 0.48
Median	4 (range 2–4)	5 (range 4–5)

## Discussion

The present study examined the diagnostic value of integrated methionine PET/MRI for suspected primary brain tumors. The study delivered two important messages. First, the number of correct diagnosis was not increased by usage of integrated methionine PET/MRI compared to MRI alone. Second, diagnostic confidence was significantly higher in integrated methionine PET/MRI compared to MRI alone. In HGG a higher diagnostic confidence would not change clinical management, whereas in LGG especially in differentiation of Astrocytoma vs. non malignant lesion the higher diagnostic confidence might change the clinical workflow towards a more radical therapy.

Staging of suspected glioma patients enables the best possible management and depends on high-quality imaging techniques. Computed tomography (CT) is the modality of choice in emergency imaging in the detection of hydrocephalus, hemorrhage or herniation. In oligodendroglial tumors an additional CT scan might be helpful to detect calcifications [17]. But MRI is the undisputed gold standard in the assessment of cerebral brain tumors [18,19], due to detailed anatomical information for surgery, radiotherapy, postoperative monitoring and for treatment assessment. Our data confirm that structural MRI is indispensible for diagnosing suspected brain tumor. Besides structural MRI multiparametric MRI (e.g. perfusion weighted imaging, diffusion weighted imaging or MR spectroscopy) was intensively assessed within the last years with controversial results for clinical routine [20,21,22].

In our collective 82.4% of the lesions were correctly identified by MRI alone, integrated methionine PET did not change the number of correct diagnoses. Our results are comparable with previous methionine PET studies, where sensitivity in diagnosing suspected gliomas was described with 76% to 100% [4,7,23], our results fit into previous studies. In other studies the role of sole methionine PET is described as controversial because there was no clear predictive value for example for grading of astrocytoma [23,24]. In clinical routine the role of the PET-component is helpful in our patient cohort because diagnostic confidence is increased. Our results demonstrate a significant difference of T/N ratios of low and high-grade astrocytoma even if there is a broad overlap. Data of methionine PET were recently described to be helpful for diagnosing low-grade glioma and might offer a prognostic biomarker in future [25]. Shinozaki et al. even found a significant increase of T/N ratio in astrocytoma as tumor grade increases and Kato et al. described a positive correlation with astrocytoma grade [26,27]. Especially in low-grade glioma the additional information of an increased methionine metabolism is helpful for evaluation [13].

To our knowledge, the enrolled number of patients in this study was the largest with suspected primary brain tumor yet studied by integrated PET/MRI. However, our results should be considered preliminary and open to further confirmation in future studies with larger cohorts. Limitations of our study should also be noted. PET-data of glioma with and without oligodendroglial component were analyzed together due to the low number of oligodendroglial tumors. Another limitation was the restricted reference standard. Histopathological sampling of all suspected lesions would be desirable to provide a reliable reference standard, however in certain lesions no clinical indication for biopsy or surgery was present. But for these not histopathologically confirmed lesions follow-up imaging was used as the reference standard. Furthermore, there was a bias in selection of patients, only those with previous MRI and suspicious and in some cases unclear findings were advised for the methionine PET/MRI. Another limitation is the PET acquisition time, which was standardized to a 20-min length but with a delay of 10–37 min after i.v. 11C-methionine injection. Although the average delay was minor (22.2 ± 4.7 min) one could assume based on previous dynamic amino acid PET studies, where low-grade glioma accumulate the tracer for a prolonged time while high-grade ones often showed an early increase followed by a decrease. This ability to separate different grades of gliomas is mostly described for studies with F-18-FET [28]. The picture is less clear with C-11-Methionine as FET as Met feature (slightly) different characteristics of transmembrane transport and intracellular distribution/metabolism. There are reports of MET exhibiting no tracer washout in high-grade tumours and that dynamic MET imaging does not allow accurate separation of low grade versus high grade gliomas [29].

To decide whether “wait and scan” or surgery is the right decision is still demanding and not yet solved. Non-enhancing lesions mimicking low-grade glioma are the challenging ones, because differentiation among stable low-grade glioma and low-grade glioma with malignant transformation is not yet possible. Watanabe et al. propose methionine PET as the diagnostic tool of choice with a cut off value of 1,9 T/N ratio [30]. In future large-scale multi-site studies are needed to investigate the diagnostic value integrated amino acid PET/MRI in glioma. Especially the integrated use of multiparametric MRI with PET is promising and offers a powerful diagnostic tool for future studies.

Moreover methionine PET is described to represent the extent of tumor more precise than CT or MRI alone [31]. The introduction of methionine PET/MRI offers a new diagnostic tool by combining two formerly separated examinations helping, amongst other advantages, to reduce investigation time. Preuss et al. showed that this leads to a reduction of associated risk factors like additional anesthesia in children, who formerly had to get anesthesia twice or prolonged by sequential MRI and PET data acquisition [32]. By identifying focal PET positive lesions, integrated methionine PET/MRI data can be used for optimal biopsy or radiotherapy planning [33]. Furthermore, the inpatient stay can be shortened, increasing patient comfort and profitability. Overall integrated methionine PET/MRI leads to an improved compliance and clinical workflow.

## Abbreviations

CT: Computed tomography
FLAIR: fluid-attenuated inversion recovery
GBM: glioblastoma multiforme
MRI: magnetic resonance imaging
PET: positron emission tomography
SWI: susceptibility weighted imaging
SUVmax: standardized uptake value of the tumor showing the maximum uptake

## References

[1] Gumprecht HK, Widenka DC, Lumenta CB (1999) BrainLab VectorVision Neuronavigation System: technology and clinical experiences in 131 cases. Neurosurgery 44: 97–104; discussion 104–105. 9894969 10.1097/00006123-199901000-00056

[2] Mabray MC, Barajas RF Jr., Cha S (2015) Modern brain tumor imaging. Brain Tumor Res Treat 3: 8–23. 10.14791/btrt.2015.3.1.8 25977902 PMC4426283

[3] Kubota K (2001) From tumor biology to clinical Pet: a review of positron emission tomography (PET) in oncology. Ann Nucl Med 15: 471–486. 11831394 10.1007/BF02988499

[4] Becherer A, Karanikas G, Szabo M, Zettinig G, Asenbaum S, Marosi C, et al (2003) Brain tumour imaging with PET: a comparison between [18F]fluorodopa and [11C]methionine. Eur J Nucl Med Mol Imaging 30: 1561–1567. 10.1007/s00259-003-1259-1 14579097

[5] Chung JK, Kim YK, Kim SK, Lee YJ, Paek S, Yeo JS, et al (2002) Usefulness of 11C-methionine PET in the evaluation of brain lesions that are hypo- or isometabolic on 18F-FDG PET. Eur J Nucl Med Mol Imaging 29: 176–182. 11926379 10.1007/s00259-001-0690-4

[6] Sasaki M, Kuwabara Y, Yoshida T, Nakagawa M, Fukumura T, Mihara F, et al (1998) A comparative study of thallium-201 SPET, carbon-11 methionine PET and fluorine-18 fluorodeoxyglucose PET for the differentiation of astrocytic tumours. Eur J Nucl Med 25: 1261–1269. 9724375 10.1007/s002590050294

[7] Nariai T, Tanaka Y, Wakimoto H, Aoyagi M, Tamaki M, Ishiwata K, et al (2005) Usefulness of L-[methyl-11C] methionine-positron emission tomography as a biological monitoring tool in the treatment of glioma. J Neurosurg 103: 498–507. 10.3171/jns.2005.103.3.0498 16235683

[8] Sadeghi N, Salmon I, Decaestecker C, Levivier M, Metens T, Wikler D, et al (2007) Stereotactic comparison among cerebral blood volume, methionine uptake, and histopathology in brain glioma. AJNR Am J Neuroradiol 28: 455–461. 17353312 PMC7977817

[9] Singhal T, Narayanan TK, Jacobs MP, Bal C, Mantil JC (2012) 11C-methionine PET for grading and prognostication in gliomas: a comparison study with 18F-FDG PET and contrast enhancement on MRI. J Nucl Med 53: 1709–1715. 10.2967/jnumed.111.102533 23055534

[10] Kobayashi K, Hirata K, Yamaguchi S, Manabe O, Terasaka S, Kobayashi H, et al (2015) Prognostic value of volume-based measurements on (11)C-methionine PET in glioma patients. Eur J Nucl Med Mol Imaging 42: 1071–1080. 10.1007/s00259-015-3046-1 25852010

[11] Delso G, Furst S, Jakoby B, Ladebeck R, Ganter C, Nekolla SG, et al (2011) Performance measurements of the Siemens mMR integrated whole-body PET/MR scanner. J Nucl Med 52: 1914–1922. 10.2967/jnumed.111.092726 22080447

[12] Quick HH (2014) Integrated PET/MR. J Magn Reson Imaging 39: 243–258. 10.1002/jmri.24523 24338921

[13] Herholz K, Holzer T, Bauer B, Schroder R, Voges J, Ernestus RI, et al (1998) 11C-methionine PET for differential diagnosis of low-grade gliomas. Neurology 50: 1316–1322. 9595980 10.1212/wnl.50.5.1316

[14] Hatakeyama T, Kawai N, Nishiyama Y, Yamamoto Y, Sasakawa Y, Ichikawa T, et al (2008) 11C-methionine (MET) and 18F-fluorothymidine (FLT) PET in patients with newly diagnosed glioma. Eur J Nucl Med Mol Imaging 35: 2009–2017. 10.1007/s00259-008-0847-5 18542957

[15] Boss A, Bisdas S, Kolb A, Hofmann M, Ernemann U, Claussen CD, et al (2010) Hybrid PET/MRI of intracranial masses: initial experiences and comparison to PET/CT. J Nucl Med 51: 1198–1205. 10.2967/jnumed.110.074773 20660388

[16] Nojiri T, Nariai T, Aoyagi M, Senda M, Ishii K, Ishiwata K, et al (2009) Contributions of biological tumor parameters to the incorporation rate of L: -[methyl-(11)C] methionine into astrocytomas and oligodendrogliomas. J Neurooncol 93: 233–241. 10.1007/s11060-008-9767-2 19099197

[17] Lee YY, Van Tassel P (1989) Intracranial oligodendrogliomas: imaging findings in 35 untreated cases. AJR Am J Roentgenol 152: 361–369. 10.2214/ajr.152.2.361 2783515

[18] van den Bent MJ, Wefel JS, Schiff D, Taphoorn MJ, Jaeckle K, Junck L, et al (2011) Response assessment in neuro-oncology (a report of the RANO group): assessment of outcome in trials of diffuse low-grade gliomas. Lancet Oncol 12: 583–593. 10.1016/S1470-2045(11)70057-2 21474379

[19] Vogelbaum MA, Jost S, Aghi MK, Heimberger AB, Sampson JH, Wen PY, et al (2012) Application of novel response/progression measures for surgically delivered therapies for gliomas: Response Assessment in Neuro-Oncology (RANO) Working Group. Neurosurgery 70: 234–243; discussion 243–234. 10.1227/NEU.0b013e318223f5a7 21593697

[20] Batra A, Tripathi RP, Singh AK (2004) Perfusion magnetic resonance imaging and magnetic resonance spectroscopy of cerebral gliomas showing imperceptible contrast enhancement on conventional magnetic resonance imaging. Australas Radiol 48: 324–332. 10.1111/j.0004-8461.2004.01315.x 15344981

[21] Fan GG, Deng QL, Wu ZH, Guo QY (2006) Usefulness of diffusion/perfusion-weighted MRI in patients with non-enhancing supratentorial brain gliomas: a valuable tool to predict tumour grading? Br J Radiol 79: 652–658. 10.1259/bjr/25349497 16641420

[22] Liu X, Tian W, Kolar B, Yeaney GA, Qiu X, Johnson MD, et al (2011) MR diffusion tensor and perfusion-weighted imaging in preoperative grading of supratentorial nonenhancing gliomas. Neuro Oncol 13: 447–455. 10.1093/neuonc/noq197 21297125 PMC3064693

[23] Yamamoto Y, Nishiyama Y, Kimura N, Kameyama R, Kawai N, Hatakeyama T, et al (2008) 11C-acetate PET in the evaluation of brain glioma: comparison with 11C-methionine and 18F-FDG-PET. Mol Imaging Biol 10: 281–287. 10.1007/s11307-008-0152-5 18543041

[24] Ceyssens S, Van Laere K, de Groot T, Goffin J, Bormans G, Mortelmans L (2006) [11C]methionine PET, histopathology, and survival in primary brain tumors and recurrence. AJNR Am J Neuroradiol 27: 1432–1437. 16908552 PMC7977552

[25] Takano K, Kinoshita M, Arita H, Okita Y, Chiba Y, Kagawa N, et al (2016) Diagnostic and Prognostic Value of 11C-Methionine PET for Nonenhancing Gliomas. AJNR Am J Neuroradiol 37: 44–50. 10.3174/ajnr.A4460 26381556 PMC7960211

[26] Shinozaki N, Uchino Y, Yoshikawa K, Matsutani T, Hasegawa A, Saeki N, et al (2011) Discrimination between low-grade oligodendrogliomas and diffuse astrocytoma with the aid of 11C-methionine positron emission tomography. J Neurosurg 114: 1640–1647. 10.3171/2010.11.JNS10553 21214332

[27] Kato T, Shinoda J, Nakayama N, Miwa K, Okumura A, Yano H, et al (2008) Metabolic assessment of gliomas using 11C-methionine, [18F] fluorodeoxyglucose, and 11C-choline positron-emission tomography. AJNR Am J Neuroradiol 29: 1176–1182. 10.3174/ajnr.A1008 18388218 PMC8118839

[28] Weckesser M, Langen KJ, Rickert CH, Kloska S, Straeter R, Hamacher K, et al (2005) O-(2-[18F]fluorethyl)-L-tyrosine PET in the clinical evaluation of primary brain tumours. Eur J Nucl Med Mol Imaging 32: 422–429. 10.1007/s00259-004-1705-8 15650870

[29] Moulin-Romsee G, D'Hondt E, de Groot T, Goffin J, Sciot R, Mortelmans L, et al (2007) Non-invasive grading of brain tumours using dynamic amino acid PET imaging: does it work for 11C-methionine? Eur J Nucl Med Mol Imaging 34: 2082–2087. 10.1007/s00259-007-0557-4 17763978

[30] Watanabe A, Muragaki Y, Maruyama T, Shinoda J, Okada Y (2016) Usefulness of (1)(1)C-methionine positron emission tomography for treatment-decision making in cases of non-enhancing glioma-like brain lesions. J Neurooncol 126: 577–583. 10.1007/s11060-015-2004-x 26612734

[31] Voges J, Herholz K, Holzer T, Wurker M, Bauer B, Pietrzyk U, et al (1997) 11C-methionine and 18F-2-fluorodeoxyglucose positron emission tomography: a tool for diagnosis of cerebral glioma and monitoring after brachytherapy with 125I seeds. Stereotact Funct Neurosurg 69: 129–135. 9711745 10.1159/000099864

[32] Preuss M, Werner P, Barthel H, Nestler U, Christiansen H, Hirsch FW, et al (2014) Integrated PET/MRI for planning navigated biopsies in pediatric brain tumors. Childs Nerv Syst 30: 1399–1403. 10.1007/s00381-014-2412-9 24710719

[33] Navarria P, Reggiori G, Pessina F, Ascolese AM, Tomatis S, Mancosu P, et al (2014) Investigation on the role of integrated PET/MRI for target volume definition and radiotherapy planning in patients with high grade glioma. Radiother Oncol 112: 425–429. 10.1016/j.radonc.2014.09.004 25308182

